# Stable incidence but increase in prevalence of ANCA-associated vasculitis in southern Sweden: a 23-year study

**DOI:** 10.1136/rmdopen-2022-002949

**Published:** 2023-03-09

**Authors:** Jens Rathmann, Mårten Segelmark, Martin Englund, Aladdin J Mohammad

**Affiliations:** 1 Clinical Sciences, Rheumatology, Lund University, Lund, Sweden; 2 Department of Nephrology, Lund University, Lund, Sweden; 3 Clinical Epidemiology Unit, Orthopedics, Department of Clinical Sciences Lund, Lund University, Lund, Sweden; 4 Department of Medicine, University of Cambridge, Cambridge, UK

**Keywords:** Systemic vasculitis, Epidemiology, Granulomatosis with polyangiitis

## Abstract

**Objective:**

To update the epidemiology of anti-neutrophil cytoplasmic antibody-associated vasculitis (AAV) in a defined geographical area of southern Sweden.

**Methods:**

The study area comprised 14 municipalities with a combined adult population (≥18 years) of 623 872 in 2019. All cases diagnosed with AAV in 1997–2019 in the study area were included in the estimate of incidence. Diagnosis of AAV was verified by case record review, and cases were classified using the European Medicines Agency algorithm. Point prevalence was estimated on 01 January 2020.

**Results:**

Three hundred and seventy-four patients (median age 67.5 years, 47% female) were diagnosed with new-onset AAV during the study period. One hundred and ninety-two were classified as granulomatosis with polyangiitis (GPA), 159 as microscopic polyangiitis (MPA) and 23 as EGPA. The average annual incidence/million adults was 30.1 (95% CI 27.0 to 33.1) for AAV: 15.4 (95% CI 13.3 to 17.6) for GPA, 12.8 (95% CI 10.8 to 14.8) for MPA and 1.8 (95% CI 1.1 to 2.6) for eosinophilic GPA (EGPA). Incidence was stable during the study period, 30.3/million 1997–2003, 30.4/million 2004–2011 and 29.5/million 2012–2019. The incidence increased with age and was highest in age group 70–84 years (96/million adults). On 1 January 2020, the prevalence was 428/million adults and was higher in males than in females (480 vs 378/million).

**Conclusions:**

The incidence of AAV in southern Sweden was found stable over the course of 23 years; while the prevalence has increased, which might indicate better management and treatment of AAV resulting in improved survival.

**WHAT IS ALREADY KNOWN ON THIS TOPIC**

- Increasing incidence and prevalence of anti-neutrophil cytoplasmic antibody (ANCA)-associated vasculitis have been described in earlier studies.

**WHAT THIS STUDY ADDS**

- Observation of stable incidence (30 cases per million) and rising prevalence (highest ever reported prevalence with 428 cases per million) in a population-based cohort with 23-year follow-up time.

**HOW THIS STUDY MIGHT AFFECT RESEARCH, PRACTICE OR POLICY**

- The incidence of ANCA-associated vasculitis (AAV) is stable during a long observation period in this large population-based cohort from Sweden, implying that changes in disease definitions and classification criteria as well as increased physician awareness and ANCA testing might explain rising incidence in earlier studies rather than true incidence increases. The prevalence is the highest ever reported in AAV, indicating improved management and, therefore, survival.

## Introduction

Anti-neutrophil cytoplasmic antibody (ANCA)-associated vasculitis (AAV) comprises a group of rare diseases that are classified according to clinicopathological characteristics into three phenotypic variants: granulomatosis with polyangiitis (GPA), microscopic polyangiitis (MPA) and eosinophilic GPA (EGPA).^1^ The disease primarily affects small blood vessels, leading to a range of pathological changes including granulomatous or necrotising inflammation in the affected organ systems. It commonly affects multiple organs, primarily lungs, kidneys and skin as well as the upper respiratory tract and the sinonasal area. Anti-neutrophil cytoplasmic antibodies, predominantly of IgG type, targeting myeloperoxidase (MPO) or proteinase-3 (PR3) can frequently be detected.^2^ AAV is associated with disease-related and/or treatment-related morbidity and mortality.^3 4^ Epidemiological studies of rare diseases such as AAV are needed to increase our understanding of aetiological mechanisms and provide a basis for the planning and allocation of healthcare resources. Common challenges in studying the epidemiology of rare diseases are differences in methods of case identification and ascertainment, small sample sizes and low incidence.^5^ Development and revision of disease definitions and classification criteria have substantially improved the comparability of findings of epidemiological studies in AAV in recent decades.^1 6 7^ Today ANCA testing is routine in diagnostic workup of AAV.^8^ MPO-AAV and PR3-AAV differ genetically^9 10^ and in disease course,^11^ and discussion of a shift from classification based on phenotype to one based on serotype is ongoing.^12 13^

The reported incidence of AAV has increased globally in recent decades.^14^ From 1998 to 2015, studies in European countries reported annual incidence of all AAV phenotypes per million inhabitants of 10.2 in northern Germany (1998–1999),^15^ 12.2 (1998–2001) in north-western Spain,^16^ 18.5 (2000–2015) in Denmark,^17^ 20.4 in the UK (1988–1997),^18^ 20.8 (1997–2006)^19^ in Sweden and 24.7 (99–2013)^20^ in Norway. Comparable incidences have been reported in Japan but with significant differences in disease phenotype and serotype distribution. Most patients were classified as MPA, with the dominating serology type being MPO-ANCA.^21 22^ The increasing incidence might be partly explained by changes in classification criteria along with increased physician awareness and availability of ANCA testing. However, in addition to geographical and demographic differences among the cited studies, methods of case identification/ascertainment and classification criteria differed, so variation and increases in incidence might be explained by factors other than true changes in the epidemiology of the disease.

Studies have reported incidences of AAV to peak in winter,^23^ summer^24^ or to show no seasonal variation.^25^

An increase in incidence, improved case identification and greater survival rates are all possible factors in rising prevalence of AAV, as reported in several studies. In the UK and northern Germany, the reported prevalence of GPA and MPA has doubled from the 1990s to 2006.^26^ Prevalence of AAV was reported as 181/million in Tromsö, Norway in 2003 compared with 352 in 2013.^20^ More recently, a prevalence rate of 421/million from Olmsted County in the USA was reported.^27^

Due to changing incidence and prevalence around the world, we present an update comparing incidence and prevalence of AAV over a 23-year period using the same case definition and classification as in our previous study.^19^ We also aim to assess possible seasonal variation in disease onset.

## Methods

### The study area

**Figure 1:**
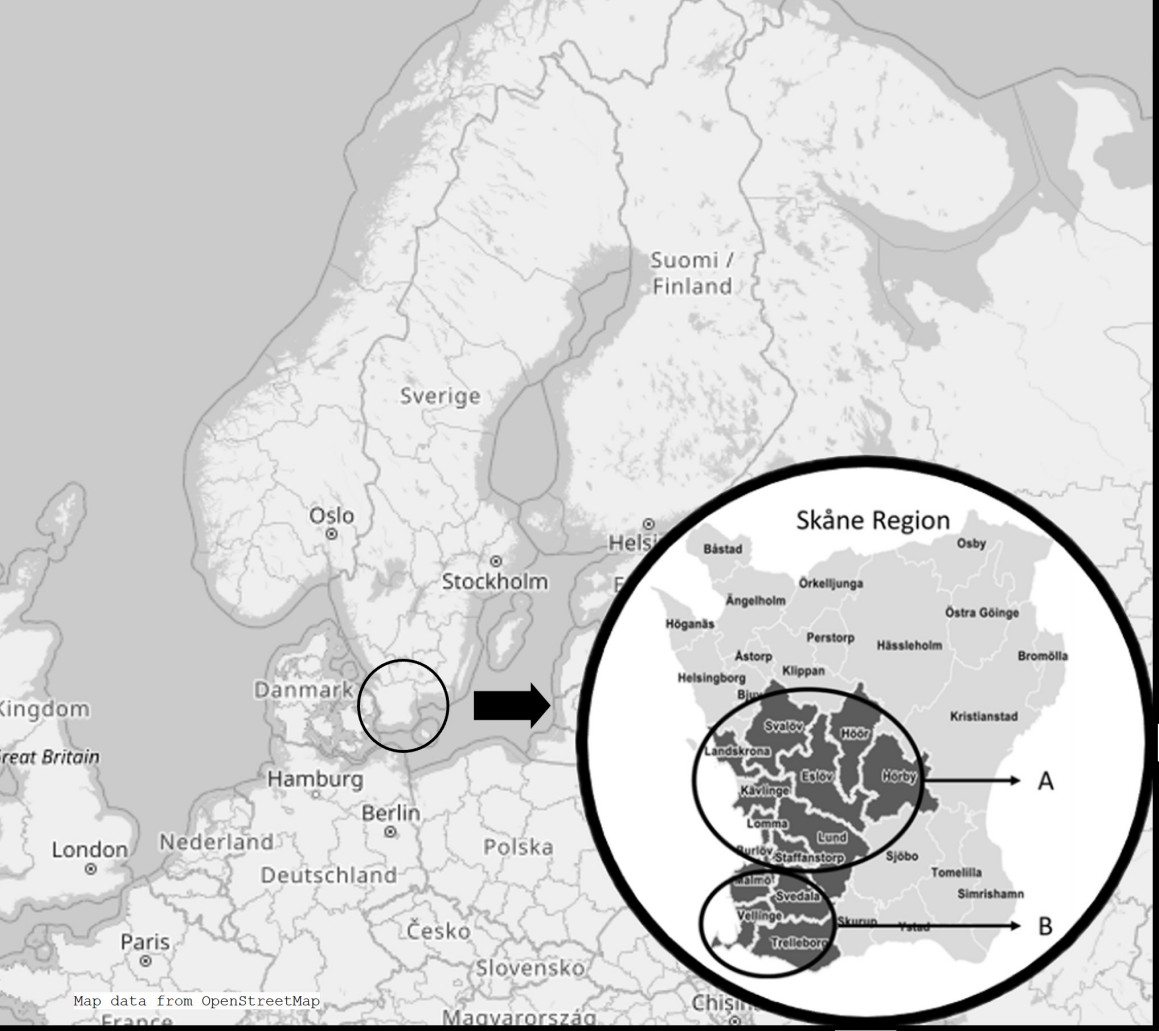
Study area A and B within the Skåne region in the southernmost part of Sweden.

The study area comprised 14 municipalities in southern Sweden. The study area includes both urban and rural areas, with a mean population density of 333/km^2^ compared with 25/km^2^ for the country as a whole. Most people live in cities. The previously described study area^19 28^ is served by four hospitals. Skåne university hospital, with campuses in Lund and Malmö, is a tertiary university hospital and referral centre for nearly 1.7 million people. Hospitals in the cities of Landskrona and Trelleborg also serve the area, both with internal medicine in-patient services. Study areas designated A and B were used for the estimates of incidence and comorbidities, and area A was used for prevalence estimates (figure 1).

### The population

The total adult population (≥18 years) was 623 872 (51% female) in December 2019. The adult population in the study area increased by 140 574 (29%) from 1997 to 2019. The age distribution in the study area was 40%, 18–39 years; 25%, 40–54; 20%, 55–69, 12%, 70–84 and 3%, >85 and was stable throughout study time.

The population structure of Sweden has undergone changes between 2000 and 2019. Specifically, the percentage of immigrants born in Asia and Africa has increased threefold. The percentage of people from Africa increased from 0.6% to 2.2%, while the percentage of people from Asia increased from 2.5% to 7.6%. In contrast, the percentage of people from other Nordic countries and the EU remained constant at around 5.5%. Immigration from non-EU Europe, Oceania and the Americas is also similar. Most immigrants are between the ages of 25 and 40.^29^

### Registries and databases

The Skåne Healthcare Register (SHR) is a comprehensive administrative register containing information regarding healthcare services in the region of Skåne, Sweden, since 1998.^30^ All healthcare contacts including visits to physicians, nurses and other healthcare professionals, along with a record of care provided, are registered under the patient’s unique 10-digit personal identity number. Diagnoses are reported in the SHR using the International Classification of Diseases, 10th revision (ICD-10). The SHR was also used to identify comorbidities in the prevalent cases.

The ANCA-serology databases at the department of clinical immunology in Lund and the private laboratory SVAR (formerly known as Wieslab AB) were used to identify patients testing positive for PR3-ANCA and MPO-ANCA.

The population registry was used to access the residential address of cases. Only cases diagnosed while living in the study area were included as incident cases. For the prevalence estimates, only patients living in the study area at date of prevalence estimate were included.

### Case retrieval

Case retrieval was slightly modified during the study period. In the first 6 years of the study,^28^ three retrieval sources (ANCA-serology, clinical and histology registries) were accessed. In our seminal study of the prevalence of primary systemic vasculitis including AAV, 80 of 86 (93%) cases were identified by combining the diagnosis registries at the Department of Nephrology and the Department of Rheumatology at Skåne University Hospital with the ANCA database.^28^ After 2003, only the diagnosis registry using the ICD-10 codes (M30.1–M31.9) and the ANCA database were used for case identification. The SHR entries for ICD10-codes M30.1–M31.9 were retrieved in 3 year increments.^30^ Similarly, the serology databases were periodically searched for persons with positive tests for either PR3-ANCA or MPO-ANCA at the two laboratories analysing ANCA serology in the study area.

### Case ascertainment and classification

Records of all potentially eligible cases identified from the retrieval sources were reviewed to confirm a diagnosis of small vessel vasculitis as defined by the European Medicines Agency (EMA) algorithm,^7^ which was also employed for disease classification. The diagnosis of small vessel vasculitis was based on symptoms compatible with, or typical of, vasculitis and supported by histological or serological findings. If biopsy was not conclusive or not performed, surrogate markers for small vessel vasculitis or granulomatous disease were used as previously described.^7^ It is also required that no other diagnosis could explain the clinical presentation. Cases fulfilling criteria were classified into AAV-phenotypes employing the EMA algorithm.^7^

### Data collection

Demographics and clinical data, including organ involvement, along with routine laboratory, serology, imaging and histology data, were collected for cases at time of diagnosis and during follow-up when available. Information of selected comorbidities (myocardial infarction, stroke, cancer, hypertension and diabetes mellitus) occurring after diagnosis of AAV was collected by linking the AAV cohort to the SHR using the corresponding ICD-10 codes for these diseases. Place of residence and vital status were acquired from the Swedish population register. End-stage renal disease (ESRD) was defined as ongoing dialysis or successful renal transplant. Information with respect to ESRD was collected from case records.

### Statistical analysis

Data are presented as frequency and percentage, mean and SD, or median with IQR when appropriate. Categorical variables were compared by using χ^2^ test. Data of study population used for the calculation of incidence and point prevalence (PP) were acquired from Statistics Sweden.^29^ For incidence estimates, all individuals newly diagnosed with AAV from 1997 to 2019 living within the study area at the time of diagnosis were included. The incidence per million adults for each calendar year during the study period was calculated using the total adult population each year on 1 January as the denominator and number of patients diagnosed in that year as the numerator. Incidences were estimated in 3-year overlapping periods (1997–1999, 1998–2000, 1999–2001, etc) to illustrate changes in incidence rate overtime. For calculation of age-specific incidence, the cohort was separated into age groups: 18–39, 40–54, 55–69, 70–84 and ≥85 years.

For study of seasonal variation, winter was defined as December–February; spring, March–May; summer, June–August; and autumn, September–November. A Poisson regression model was used to estimate seasonal incidence ratios, with winter as reference.

We estimated the prevalence at four time points, 2003, 2010, 2015 and 2020, with the pp per million adults estimated on 1 January 2020 as a measure of overall prevalence. To allow follow-up and comparison of prevalence figures in southern Sweden over time, we selected pp data only from study area A, which was the site of our first report in 2007^28^ (figure 1). Data from the earlier study were recalculated to reflect only prevalence in the adult population.^28^ The denominator of prevalence estimate was adult population at pp dates. The numerator was the number of prevalent cases living in study area A on the corresponding date. A normal approximation method was used for the calculation of CIs for incidence and prevalence estimates. To give a relative measure of disease burden on the pp date, selected comorbidities diagnosed after the onset of AAV were identified and presented as number and percentage. All statistical calculations were conducted with Statistical Package for the Social Sciences, SPSS V.26 for Windows (IBM SPSS Statistics).

## Results

### Patients

Three hundred and seventy-four cases (47% female) were diagnosed with AAV during the 23-year study period. The median age at diagnosis was 67.5 (IQR 55–77) years for all patients, 68 (IQR 56.2–77) for males and 67 (IQR 53–77.2) for females. One hundred and ninety-two patients were classified as GPA, 159 as MPA and 23 as EGPA. One hundred and eighty-eight were PR3-ANCA positive, 161 were MPO-ANCA positive and 25 patients were ANCA negative. Patients diagnosed with MPA were older at time of diagnosis (median age 72 (IQR 60–81)) than those with GPA (median age 64 (IQR 52–73)) and EGPA (median age 60 (IQR 37–63)).

### Incidence

**Figure 2:**
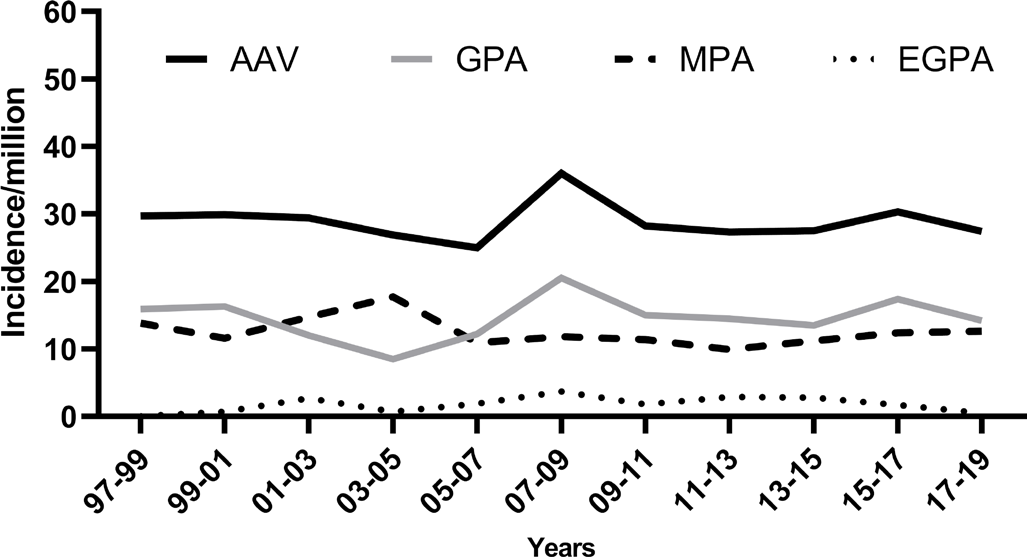
Incidence of AAV per million adults, stratified by 3 years overlapping, for all AAV and different phenotypes. AAV, anti-neutrophil cytoplasmic antibody-associated vasculitis; EGPA, eosinophilic granulomatosis with polyangiitis; GPA, granulomatosis with polyangiitis; MPA, microscopic polyangiitis.

The calculated mean annual incidence 1997–2019 for AAV was 30.1 per million adults (95% CI 27.0 to 33.1) for all AAV, 15.4 (95%CI 13.3 to 17.6) for GPA, 12.8 (95%CI 10.8 to 14.8) for MPA and 1.8 (95%CI 1.1 to 2.6) for EGPA. The incidence of AAV was higher in males, 32.9 (95% CI 28.4 to 37.5) than in females 27.3 (95% CI 23.3 to 31.4) (table 1). The mean annual incidence from 1997 to 2003 was 30.3 per million (95% CI 24.4 to 36.1), 30.4 per million (95% CI 25.2 to 35.7) 2004–2011 and 29.5 per million (95% CI 24.6 to 34.4) from 2012 to 2019. The incidence was estimated in 3-year overlapping periods and was relatively stable throughout the study period (figure 2).

**Table 1:** Average annual incidence per million adults for AAV based on disease phenotype and ANCA serology

	AAV n=374 (95% CI)	GPA n=192 (95% CI)	MPA n=159 (95% CI)	EGPA n=23 (95% CI)	PR3 + n=188 (95% CI)	MPO + n=161 (95% CI)
All	30.1 (27.0 to 33.1)	15.4 (13.3 to 17.6)	12.8 (10.8 to 14.8)	1.8 (1.1 to 2.6)	15.0 (12.9 to 17.2)	12.9 (10.9 to 14.9)
Male	32.9 (28.4 to 37.5)	18.3 (14.9 to 21.7)	13.2 (10.3 to 16.1)	1.5 (0.5 to 2.4)	18.8 (15.3 to 22.2)	12.2 (9.4 to 15.0)
Female	27.3 (23.3 to 31.4)	12.7 (10.0 to 15.5)	12.4 (9.7 to 15.1)	2.2 (1.0 to 3.4)	11.5 (8.8 to 14.1)	13.7 (10.8 to 16.5)

ANCA-negatives (n = 25) not shown.

AAV, ANCA-associated vasculitis; ANCA, anti-neutrophil cytoplasmic antibody; EGPA, eosinophilic granulomatosis with polyangiitis; GPA, granulomatosis with polyangiitis; MPA, microscopic polyangiitis; MPO, myeloperoxidase; PR3, proteinase-3.

### Age-specific and sex-specific incidence

**Figure 3:**
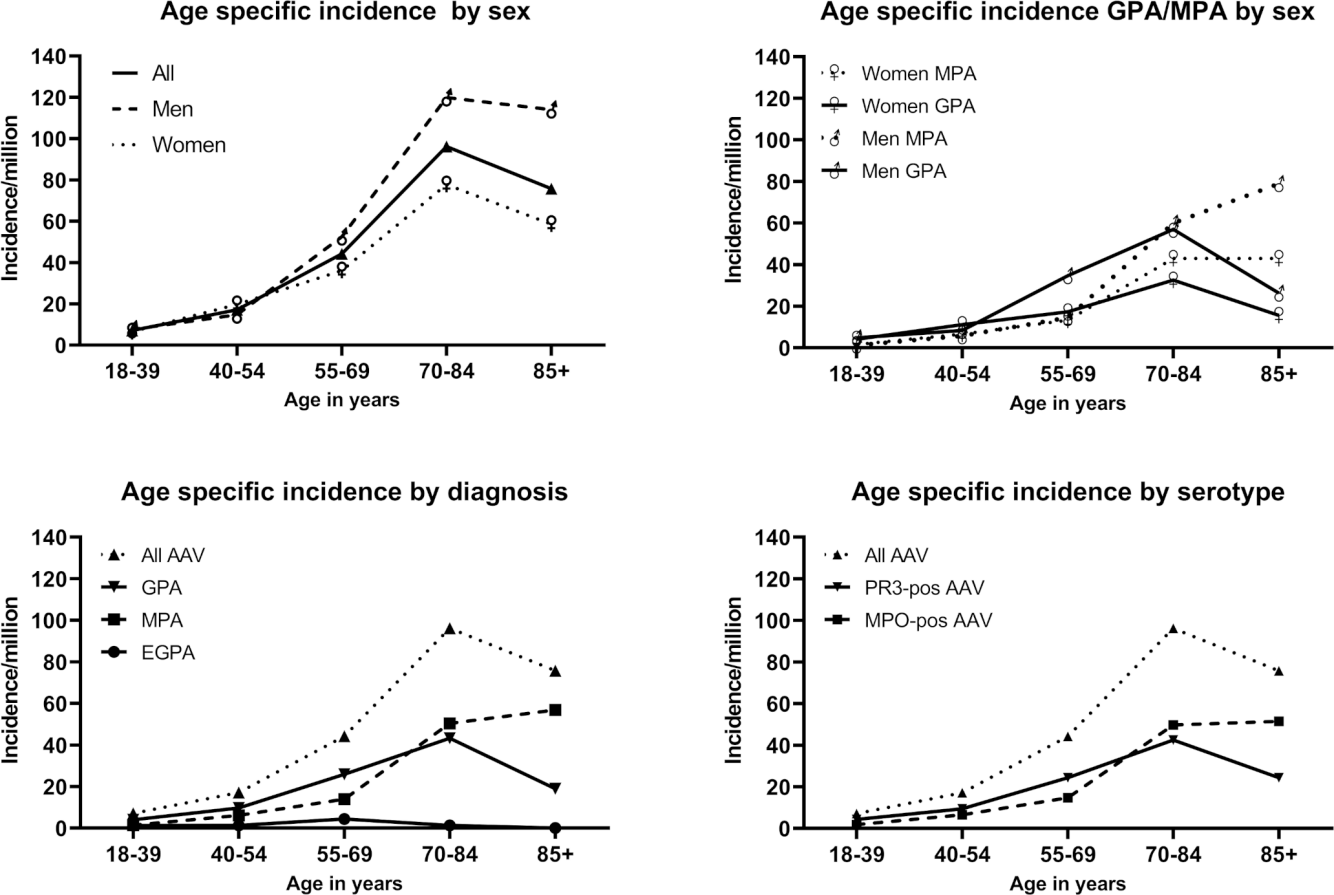
Age specific incidence rates in respect to phenotype, serology and sex. AAV, anti-neutrophil cytoplasmic antibody-associated vasculitis; EGPA, eosinophilic granulomatosis with polyangiitis; GPA, granulomatosis with polyangiitis; MPA, microscopic polyangiitis.

The incidence of AAV increased with age. The highest incidence was in the age group 70–84 years: 96.2 per million adults (95% CI 80.6 to 111.7) overall, 77.8 per million (95% CI 59.2 to 96.4) females and 119.9 per million (95% CI 93.6 to 146.0) males (figure 3). Highest GPA incidence rates were also observed in those 70–84 years old (43.2 per million) and decreased in the age group ≥85 years to 18.9 per million. The peak incidence of MPA was observed in the age group ≥85 years at 56.8 per million. This age-related increase of MPA incidence could be attributed to males, in whom we observed an increase from 59.9 per million (70–84 years) to 78.9 per million (≥85). In females, incidence was similar in these age groups (43 per million). Highest incidence of EGPA was observed in those 55–69 years (4.4 per million) (figure 3). Age-specific incidence by serotype was nearly identical to phenotype distribution (figure 3).

### Seasonal variation

**Figure 4:**
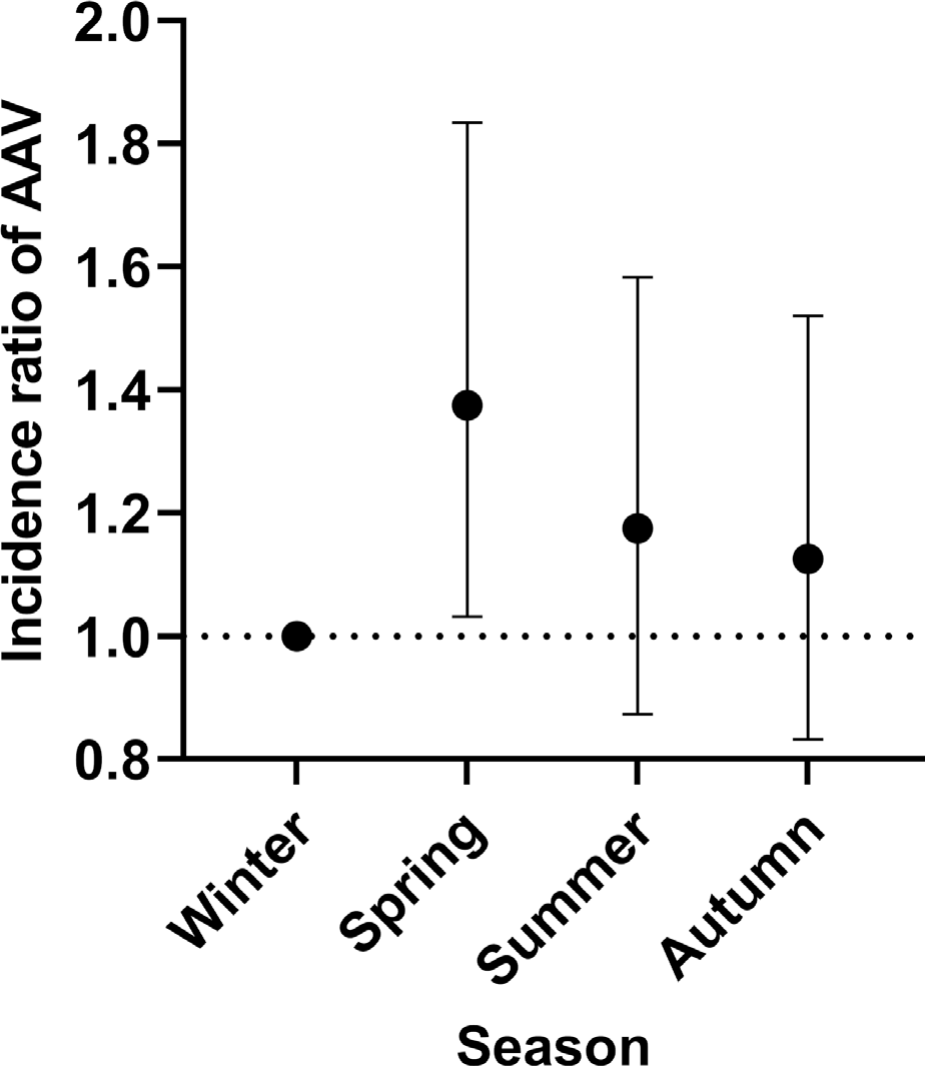
Seasonal variations. Incidence ratios with 95% CI of AAV-diagnoses in seasons (winter is reference). AAV, anti-neutrophil cytoplasmic antibody-associated vasculitis.

The seasonal distribution of AAV diagnosis was 80 cases (21%) in winter, 110 (29%) in spring, 94 (25%) in summer and 90 (24%) in autumn. The greatest number of cases were diagnosed in March and May (both n=37), followed by April (n=36). With winter as reference, a significantly higher incidence ratio (IR) was observed in spring (IR 1.38 (95% CI 1.03 to 1.83)) for all AAV (figure 4), 1.43 (95% CI 0.90 to 2.14) for GPA and 1.45 (95% CI 0.90 to 2.26) for MPA. EGPA showed higher numbers in winter. The incidence ratios for spring with winter as a reference were 1.27 (95% CI 0.80 to 1.91) for PR3-positive disease and 1.59 (95% CI 1.03 to 2.48) for MPO-positive disease.

### Point prevalence

Data of prevalence prior to the study period (1996 and earlier) were only available for 10 of the 14 municipalities in the study area with a total adult population of 273 135 (designated study area A). In area A, 50 patients were diagnosed with AAV prior to 1997. Twenty-five of these were still living in the study area at pp 1 January 2020, along with a further 92 incident cases, resulting in 117 patients included in 2020 prevalence estimates. The prevalence of AAV on 1 January 2020 was 428.4 per million adults (95% CI 350.7 to 506.0) for all patients (table 2), 241.6 (95% CI 183.3 to 299.9) for GPA, 150.1 (95% CI 104.2 to 196.1) for MPA and 36.6 (95% CI 13.9 to 59.3) for EGPA. The median age at pp 2020 was 71.5 (IQR 60.1–78.2) for all patients, 71.1 (IQR 56.1–75.7) for GPA, 74.2 (IQR 65.1–81.8) for MPA and 65.5 (IQR 52.1–79.5) for EGPA. The sex-specific prevalence was 377.8 (95% CI 275.1 to 480.5) in women and 479.7 (95% CI 363.1 to 596.4) in men. In comparison, prevalence for all AAV in 2003 was recalculated using the adult population as denominator and was 353.6 per million adults (95% CI 275.6 to 431.6). The prevalence at four time points is shown in table 2.

**Table 2:** Point prevalence (PP) of ANCA-associated vasculitis (AAV) per million adults

	N	AAV pp (95% CI)	n	GPA pp (95% CI)	n	MPA pp (95% CI)	n	EGPA pp (95% CI)
2003	79	353.6 (275.6 to 431.6)	49	219.3 (157.9 to 280.7)	24	107.4 (64.4 to 150.4)	6	26.9 (5.4 to 48.32)
2010	107	434.7 (352.3 to 517.1)	58	235.6 (175.0 to 296.3)	40	162.5 (112.1 to 212.9)	9	36.6 (12.7 to 60.5)
2015	121	468.9 (385.3 to 552.4)	68	263.5 (200.9 to 326.1)	42	162.8 (113.5 to 212.0)	11	42.6 (17.4 to 67.8)
2020	117	428.4 (350.7 to 506.0)	66	241.6 (183.3 to 299.9)	41	150.1 (104.2 to 196.1)	10	36.6 (13.9 to 59.3)

The total adult population in study area A increased from 223 419 in 2003 to 273 135 in 2020.

ANCA, anti-neutrophil cytoplasmic antibody; EGPA, eosinophilic granulomatosis with polyangiitis; GPA, granulomatosis with polyangiitis; MPA, microscopic polyangiitis.

### Comorbidities

On 01 January 2020, 220 patients with AAV were living in the study area. The most common comorbidity was hypertension (50%, n=110), followed by cancer (27%, n=59) and diabetes (19.5%, n=43). At pp 2020, 135 patients (61%) had suffered at least one comorbidity and 86 (39%) had suffered two comorbidities. Online supplemental table S1 lists comorbidities diagnosed in prevalent cases following diagnosis of AAV.

10.1136/rmdopen-2022-002949.supp2 Supplementary data

## Discussion

We report the incidence and prevalence of AAV in southern Sweden using a well-defined population-based cohort diagnosed over a period of 23 years. Incidence of AAV was relatively stable throughout the study period. Incidence increased with age and showed seasonal variation, with a higher number of patients diagnosed during spring. We observed an increase in prevalence during the study period with, to the best of our knowledge, highest prevalence ever reported for AAV.

We used similar retrieval sources, case identification and classification system throughout the 23-year study period, eliminating other variables that might explain the observed stability of incidence. A previous Swedish study relying on ICD-10 codes in a national registry demonstrated progressive increase in incidence of GPA from the early 1970s–2001.^31^ Watts *et al*^18^ similarly describe an increase in a 10-year study in Norwich, UK, as do Takala *et al*^32^ in Finland. A study from Taiwan^33^ also reports rising incidence. The most likely source of this apparent increase in incidence is the introduction of ANCA testing in the mid-1980s and early 1990s,^34^ the publication of ACR 1990 classification criteria of GPA and EGPA,^6 35^ and the CHCC 1994 definitions of vasculitis.^36^ Our results suggest that AAV incidence may have been stable during this time period and the observed differences may be attributed to changes in diagnosis and classification of AAV rather than a genuine increase in the incidence. Our results are in agreement with the most recently reported studies from Norway^20^ and the USA.^27^

Earlier findings of latitudinal differences, the so called ‘north-south gradient’ in the relative incidence of GPA and MPA^37^ are not evident in this cohort, as incidences of GPA and MPA are similar, which is in line with our earlier findings.^19^ The incidence of EGPA is comparable to reported data from the UK, 2.7 per million (95% CI 1.3 to 4.8),^18^ Australia, 2.2 per million (95% CI 0.6 to 7.2)^38^ and the USA, 4 per million (95% CI 1.0 to 6.0).^27^

The incidence of AAV increases with age. Peak incidence was at 70–84 years. The peak age at disease onset differs with disease phenotype, and was 70–84 years for GPA, while incidence of MPA peaks in the ≥85 years age group. The peak age at onset was greater than reported in previous studies of primary systemic vasculitis and GPA from the UK,^18^ Finland^32^ and Norway,^39^ where incidence peaked in the age group 65–74 years and in Spain where it was 55–64 years.^16^ Pearce *et al* reported a peak age at diagnosis of≥85 years^40^ in a study from the UK including patients with AAV diagnosed from 2007 to 2013. When we reanalysed our cohort including only patients diagnosed 2007–2019, we observed similar findings, with highest incidence in the age group ≥85 years (online supplemental figure S2). This finding might indicate a shift towards older age at diagnosis possibly related to increased physician awareness and the widespread availability and use of ANCA testing, regardless of age or comorbidity burden.

10.1136/rmdopen-2022-002949.supp1 Supplementary data

We observed highest incidence of AAV diagnosis in spring. Several studies have discussed seasonal variation in incidence of AAV, but findings are contradictory. Falk *et al*^41^ reported highest incidence in winter for all AAV. A French study of GPA found highest incidence in summer,^42^ others found no seasonal variation.^43^ Differences in study design, case definition and data collection might explain these differences and limit comparability.

Studying seasonality at diagnosis of AAV aims to shed light on etiological aspects of allergic or infectious triggers.^42 44^ The result of seasonal analysis in this study supports the recently published data from our group demonstrating increased risk of AAV following infections, especially those affecting the respiratory tract.^44^ A plausible pathophysiologic mechanism is that respiratory infection during winter may trigger the onset of AAV a few months later. A recent study from Scotland of GPA and MPA did not show seasonality,^25^ but comparability is limited because of differences in study population. Patients in the study were recruited via a renal biopsy register, and the fraction of MPA patients was much higher, not representative of the entire spectrum of AAV as is our population-based cohort.

The prevalence of AAV in our area increased from 2003 to 2015 and slightly decreased after 2015. The increase in prevalence despite stable incidence might be attributed to improvements in patient survival, physician awareness and treatment. During the study period, there was a shift to less toxic treatment regimens,^45^ as well the use of biological therapy. Studies of PP aim to assess burden of disease in a population to scale the dimension of future healthcare resources needed. In addition to the challenges that increased AAV prevalence implies, these patients are more prone to comorbidities.^46^

Strengths of this study include the population-based setting, with no referral or selection bias, and the inclusion of only patients with confirmed clinical diagnosis of small vessel vasculitis. In addition, all were classified using the same algorithm over a long period of time. This study includes a large population-based cohort encompassing the entire spectrum of AAV from mild disease to severe multisystemic vasculitis with continuous inclusion of patients using similar case identification criteria. A further strength is a validated search strategy with high case identification and completeness and robust case ascertainment. The long study period enabled us to assess incidence over time as well as seasonal variation of the disease.

The incidence of AAV in southern Sweden was found stable over the course of 23 years; while the prevalence has increased, which might indicate better management and treatment of AAV resulting in improved survival.

## Data Availability

All data relevant to the study are included in the article or uploaded as online supplemental information.
