# Supplementary material for: Stable incidence but increase in prevalence of ANCA-associated vasculitis in southern Sweden: a 23-year study
Source: RMD Open. 2023 Mar 9;9(1):e002949. doi: 10.1136/rmdopen-2022-002949 (PMC10008447; doi:10.1136/rmdopen-2022-002949)
Supplement: Supplementary data [file rmdopen-2022-002949supp002.pdf]

Table S1 Comorbidities occurring post–AAV diagnosis, pp 01 January 2020

Patients alive on 01 January 2020	**All n = 220 n (%)**	**Male n = 116 n (%)**	**Female n = 104 n (%)**	**GPA n = 133 n (%)**	**MPA n = 66 n (%)**
Myocardial infarction (MI)	21 (9.5)	16 (13.8)	5 (4.8)	14 (10.5)	6 (9.1)
Stroke	24 (10.9)	15 (12.9)	9 (8.7)	17 (12.8)	5 (7.6)
MI or stroke	41 (18.6)	27 (23.3)	14 (13.5)	27 (20.3)	11 (16.7)
Cancer	59 (26.8)	33 (28.4)	26 (25.0)	36 (27.1)	18 (27.3)
Hypertension	110 (50.0)	59 (50.9)	51 (49.0)	64 (48.1)	40 (60.6)
Diabetes mellitus	43 (19.5)	22 (19.0)	21 (20.2)	30 (22.6)	10 (15.2)
ESRD	13 (5.9)	7 (6.0)	6 (5.8)	4 (1.5)	9 (13.6)

EGPA not shown. ESRD: End stage renal disease
