# Supplementary figures and images for: Stable incidence but increase in prevalence of ANCA-associated vasculitis in southern Sweden: a 23-year study
Source: RMD Open. 2023 Mar 9;9(1):e002949. doi: 10.1136/rmdopen-2022-002949 (PMC10008447; doi:10.1136/rmdopen-2022-002949)

Age specific incidence by sex

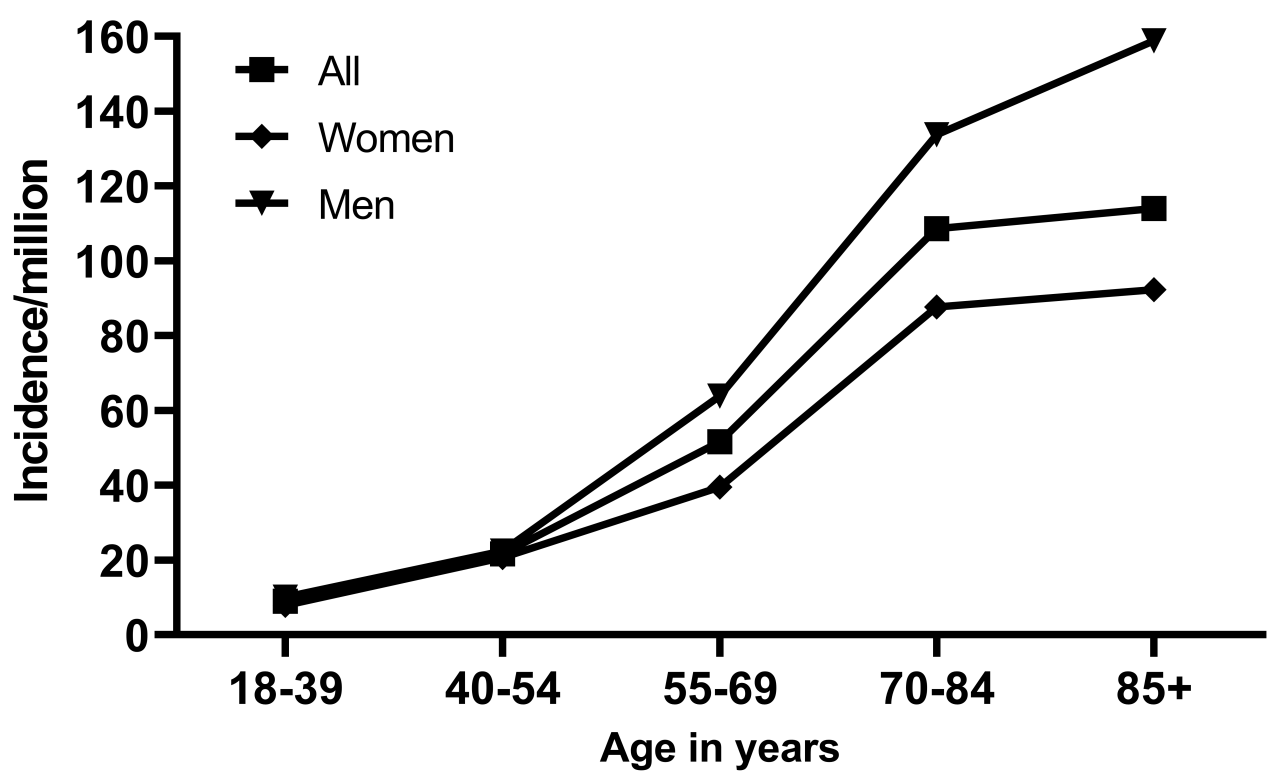

Supplement: Supplementary data [file rmdopen-2022-002949supp001.pdf]
